# Physiological Role of ATPase for GABA_A_ Receptor Resensitization

**DOI:** 10.3390/ijms23105320

**Published:** 2022-05-10

**Authors:** Sergey A. Menzikov, Danila M. Zaichenko, Aleksey A. Moskovtsev, Sergey G. Morozov, Aslan A. Kubatiev

**Affiliations:** 1 Institute of General Pathology and Pathophysiology, Russian Academy of Sciences, 8, Baltiyskaya St., 125315 Moscow, Russia; danilamihailovich@mail.ru (D.M.Z.); bioinf@mail.ru (A.A.M.); biopharm@list.ru (S.G.M.); aslan.kubatiev@gmail.com (A.A.K.); 2 Russian Medical Academy of Postdoctoral Education, Federal State Budgetary Educational Institution of Further Professional Education of the Ministry of Healthcare of the Russian Federation, 2/1, Barrykadnaya St., 125993 Moscow, Russia

**Keywords:** rat, cortical synaptoneurosomes, GABA_A_R, chloride, bicarbonate, desensitization, resensitization, ATPase, β3 subunit, transport

## Abstract

γ-Aminobutyric acid type A receptors (GABA_A_Rs) mediate primarily inhibitory synaptic transmission in the central nervous system. Following fast-paced activation, which provides the selective flow of mainly chloride (Cl^−^) and less bicarbonate (HCO_3_^−^) ions via the pore, these receptors undergo desensitization that is paradoxically prevented by the process of their recovery, referred to as resensitization. To clarify the mechanism of resensitization, we used the cortical synaptoneurosomes from the rat brain and HEK 293FT cells. Here, we describe the effect of γ-phosphate analogues (γPAs) that mimic various states of ATP hydrolysis on GABA_A_R-mediated Cl^−^ and HCO_3_^−^ fluxes in response to the first and repeated application of the agonist. We found that depending on the presence of bicarbonate, opened and desensitized states of the wild or chimeric GABA_A_Rs had different sensitivities to γPAs. This study presents the evidence that recovery of neuronal Cl^−^ and HCO_3_^−^ concentrations after desensitization is accompanied by a change in the intracellular ATP concentration via ATPase performance. The transition between the desensitization and resensitization states was linked to changes in both conformation and phosphorylation. In addition, the chimeric β3 isoform did not exhibit the desensitization of the GABA_A_R-mediated Cl^−^ influx but only the resensitization. These observations lend a new physiological significance to the β3 subunit in the manifestation of GABA_A_R resensitization.

## 1. Introduction

As members of the pentameric ligand-gated ion channels family (pLGIC), γ-Aminobutyric acid type A receptors (GABA_A_Rs) are mainly involved in inhibition but also in excitation of the central nervous system of vertebrates under certain circumstances [1,2]. Upon mediator binding, GABA_A_Rs quickly open their transmembrane pore to enable the passive flow of chloride (Cl^−^) and less bicarbonate (HCO_3_^−^) ions via the plasma membrane. However, following activation, GABA_A_Rs undergo desensitization, which involves inchmeal entry into the long-lived closed state refractory to excessive activation [3,4,5,6]. Although the causes and the roles of receptor desensitization continue to be debated, they potentially include the reduction in responses during high-frequency neurotransmitter release and the prolongation of synaptic currents [7,8]. In addition, the GABA_A_R desensitization is extremely related to the slowdown of their deactivation, called resensitization [9,10]. The linkage between desensitization and resensitization of GABA_A_R-mediated currents is demonstrated in a response to a prolongation [2,5] or reapplication of GABA [3,11]. However, despite the critical importance of GABA_A_R resensitization in maintaining their responsiveness to subsequent activation, it is unclear which molecular events trigger this long-term receptor alteration.

The regulation of desensitization and resensitization of GABA_A_Rs is an important mechanism controlled at the receptor level and within the signaling pathway, but regulation of the receptors assumes great significance in understanding the withdrawal of neurological disorders [5]. In addition, the regulation of GABA_A_R neurotransmission plays an exceptional role in psychiatric disabilities and addiction [12,13]. Many studies of desensitization have focused on changes to the receptor following massive activation with agonists or allosteric ligands that affect the occupancy of this state during synaptic inhibition [9,14]. Such changes can include phosphorylation of subunits by kinases [5], expression, clustering [15], and receptor pharmacology [16]. Additionally, some structural works have shown that the differences between the agonist-bound open (conducting) state and desensitization (hindering) state are conformational changes occurring at the “internal face” of the receptor. Recent functional and structural studies provide compelling evidence for a “dual-gate” model, in which the transmembrane domain (TMD) of pLGICs contains both an activation gate, located in the upper half of the channel, and a de-gate, at the intracellular end of the channel [17,18].

Preliminary studies have shown that the GABA_A_R-mediated current is significantly elevated by inhibitors of phosphatases [19,20] or non-hydrolysable ATP analogues [21], indicating the involvement of the processes of phosphorylation and dephosphorylation in regulating GABA_A_R functional activity [22]. It has also been demonstrated during intensive agonist action that the diminution in intracellular adenosine triphosphate concentration ([ATP]_i_) is the main reason underpinning the functional rundown of GABA_A_R function, eventually paving the way for neuronal excitation [23,24,25,26]. However, the molecular determinants that provide the ATP depletion within the GABA_A_R rundown phenomenon remain poorly understood. Recently, we reported that the GABA_A_R β3 subunit possesses Cl^−^ and HCO_3_^−^ ATPase activity, which provides the anion homeostasis and is involved in neurological disorders [27,28]. Given that such an ATPase is specifically regulated by GABAergic agonists or antagonists [27] and the ATP-hydrolyzing center is localized at the intracellular side of the channel [29], we set out to test the hypothesis about its participation in the processes of the desensitization and resensitization. Specifically, our focus was on γ-phosphate-modified ATP analogues (γPAs) that are often used to mimic various important states of ATP hydrolysis: non-hydrolysable ATP analogue adenylyl-imidodiphosphate (AMP-PNP)—the pre-hydrolytic state, and vanadate (VO_4_^3−^) and fluoride metal (AlF_x_)—the transition state [30]. We found that activation (open) and desensitization states of GABA_A_R-mediated Cl^−^ and HCO_3_^−^ flows in the synaptoneurosomes (SNs) of the cortical neurons of the rat brain were different in terms of the sensitivity to γPAs. The transition between the desensitization and resensitization states was correlated with the changing intracellular concentration of ATP ([ATP]_i_) determined by HCO_3_^−^. The β3 subunit was established as being essential for the process of GABA_A_R resensitization by ATPase performance. By using the thiol-modifying agent n-ethylmaleimide (NEM), we provided evidence of differences in the conformational properties of activation and desensitization states of GABA_A_Rs. The important role of conformational rearrangements and the resulting changes in the processes of phosphorylation and dephosphorylation was confirmed by using a mutant receptor that substantially alters the receptor’s ability to access the transition from a desensitized state to a resensitized state.

## 2. Results

### 2.1. Reapplication of GABA Causes Desensitization of GABA_A_R-Mediated Cl^−^ Influx

**Figure 1:**
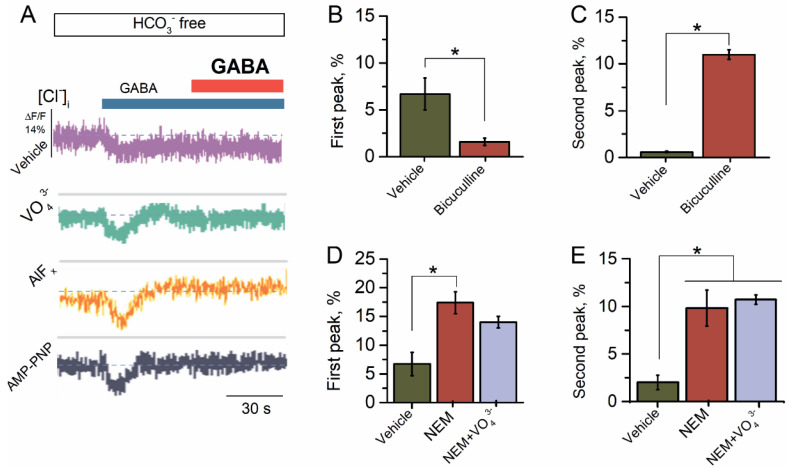
Reapplication of GABA causes a desensitization of GABA_A_R-mediated Cl^−^ influx. (**A**) Representative images of MQAE fluorescence changes in SNs recorded in response to the first application of GABA (100 μM) and reapplication of GABA (100 μM) in the HCO_3_^−^-free experimental medium, without or in the presence of VO_4_^3^^−^ (20 μM), AlF_x_ (20 μM), or AMP-PNP (2 mM). (**B**) Bar graph of percentage in MQAE peak fluorescence changes in response to the first application of GABA (100 μM), without or in the presence of bicuculline (50 μM) in an experimental medium (one-way ANOVA, Tukey’s test: F_(19)_ = 6.7, *p* = 0.005, *n* = 5). (**C**) Bar graph of percentage in MQAE peak fluorescence changes in response to reapplication of GABA (100 μM), without or in the presence of bicuculline (50 μM) in an experimental medium (one-way ANOVA, Tukey’s test: F_(19)_ = 142.7, *p* = 0.0000025, *n* = 5). (**D**) Bar graph of percentage in MQAE peak fluorescence changes in response to first application of GABA (100 μM) without or containing NEM (300 μM) in an experimental medium, in the absence or presence of VO_4_^3−^ (20 μM) (one-way ANOVA, Tukey’s test: F_(19)_ = 16.4, *p* = 0.005 or F_(19)_ = 23.8, *p* = 0.005, *n* = 5), respectively. (**E**) Bar graph of percentage in MQAE peak fluorescence changes in response to reapplication of GABA (100 μM) without or containing NEM (300 μM), in the absence and presence of VO_4_^3−^ in an experimental medium (20 μM) (one-way ANOVA, Tukey’s test: F_(19)_ = 9.1, *p* = 0.05 or F_(19)_ = 106.2, *p* = 0.00005, *n* = 5). All data in the bar graphs in this figure and those proceeding it are presented as mean values +/− SEM; * *p* < 0.05.

The preliminary studies have shown that the GABA_A_Rs of the SNs provide the Cl^−^ influx via plasma membranes [31,32,33]. Earlier, we showed that GABA (1–100 μM) increased the ATPase activity of the ternary (α2β3γ2) GABA_A_R subtype with maximum effect at 100 μM [27]. To explore the GABA_A_R-mediated Cl^−^ influx, the SNs initially were loaded with dye for Cl^−^ (MQAE) and then exposed to 10, 50, or 100 μM of GABA. The SNs in the HCO_3_^−^-free medium showed a Cl^−^ influx in response to the application of GABA with a maximum peak of fluorescence changes of 2.6 ± 0.3%, 4.4 ± 0.3%, or 6.7 ± 0.2%, respectively. Therefore, as illustrated in Figure 1A, we used GABA at a concentration of 100 μM. Some studies have shown that the repetitive application of the same agonist concentration can induce the desensitization of the GABA-evoked Cl^−^ current [11]. We applied second applications of the agonist to assess the nature of agonist-dependent desensitization and resensitization of GABA_A_R. However, reapplication of GABA (100 μM) revealed apparent desensitization of the GABA_A_R-mediated Cl^−^ influx that is similar to literature data. Specifically, the studies showed that in rat central neurons, the GABA_A_R-mediated Cl^−^ current leads to substantial desensitization with the repetitive application of GABA [11].

In exploring and establishing the role of the ATP-hydrolyzing system in the desensitization and resensitization, we studied the effect of VO_4_^3−^ (20 μM), AlF_x_ (20 μM), or AMP-PNP (2 mM) on the GABA_A_R-mediated Cl^−^ influx. All test γ-PAs caused an increase in the GABA-mediated Cl^−^ influx into neurons by approximately two times at first and had no effect on the second application of the mediator (Figure 1A). Bicuculline completely suppressed the first peak of the GABA-mediated fluorescence changes, indicating the receptor-dependent method of mediator action (Figure 1B). By contrast, at the second application of the mediator, bicuculline significantly activated the Cl^−^ influx in the SNs with a maximum peak of fluorescence changes of 11.0 ± 0.5% (*n* = 5) (Figure 1C).

It has been shown that synaptic sulfhydryl groups of ionic channels and transporters are targets for electrophiles [34,35]. Specifically, NEM has been used as an agent to regulate the cation–chloride cotransporter (CCC) function by modulating their phosphorylation [35]. In addition, NEM (300 μM) causes an increase in the frequency of the GABA_A_R-mediated postsynaptic currents [36,37] and eliminates the depolarization- or post-burst-induced suppression of GABA_A_R-mediated inhibition in CA1 pyramidal cells [38,39]. Here, NEM (300 μM) activated both at first and at the reapplication of GABA with a maximum peak of fluorescence changes of 17.4 ± 1.9% (*n* = 5) and 9.8 ± 1.9% (*n* = 5), respectively (Figure 1D,E). NEM effect did not change significantly amid the presence of VO_4_^3−^ (20 μM) in the first case and reapplication of the agonist (Figure 1D,E).

### 2.2. Bicarbonate Determines a Transition from Desensitization to Resensitization State of GABA_A_R-Mediated Cl^−^ Influx

**Figure 2:**
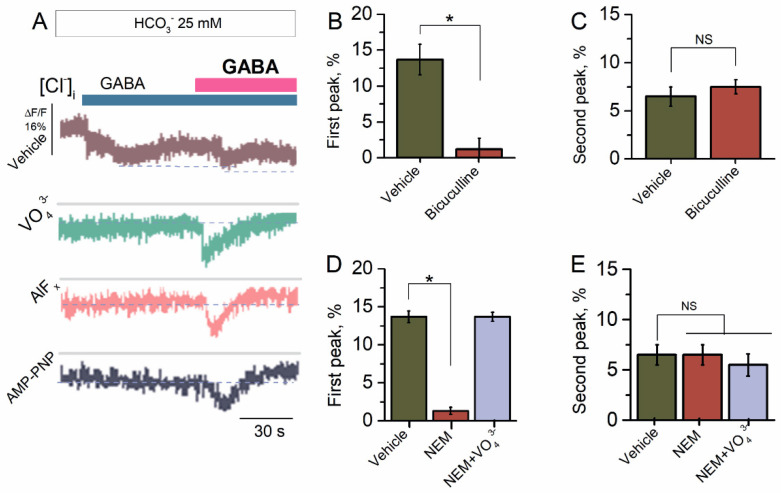
Bicarbonate hindered the desensitization of GABA_A_R-mediated Cl^−^ influx. (**A**) Representative images of MQAE fluorescence changes in SNs recorded in response to first application of GABA (100 μM) and reapplication of GABA (100 μM) in the presence of HCO_3_^−^ (25 mM) in an experimental medium, without or in the presence of VO_4_^3−^ (20 μM), AlF_x_ (10 μM), or AMP-PNP (2 mM). (**B**) Bar graph of percentage in MQAE peak fluorescence changes in response to first application of GABA (100 μM), without or in the presence of bicuculline (50 μM) (one-way ANOVA, Tukey’s test: F_(6,9)_ = 47.5, *p* = 0.00075, *n* = 7). (**C**) Bar graph of percentage in MQAE peak fluorescence changes in response to reapplication of GABA (100 μM), without or in the presence of bicuculline (50 μM) (one-way ANOVA, Tukey’s test: F_(6,9)_ = 1.5, *p* = 0.05, *n* = 7). (**D**) Bar graph of percentage in MQAE peak fluorescence changes in response to first application of GABA (100 μM) without or containing NEM (300 μM), in the absence or presence of VO_4_^3−^ (20 μM) in an experimental medium (one-way ANOVA, Tukey’s test: F_(9,6)_ = 23.7, *p* = 0.0005, *n* = 6 or F_(9,6)_ = 0.01, *p* = 0.05, *n* = 6). (**E**) Bar graph of percentage in MQAE peak fluorescence changes in response to reapplication of GABA (100 μM) without or containing NEM (300 μM), in the absence or presence of VO_4_^3−^ (20 μM) in an experimental medium (one-way ANOVA, Tukey’s test: F_(9,6)_ = 0.13, *p* = 0.05, *n* = 6 or F_(9,6)_ = 0.4, *p* = 0.05, *n* = 6). All data in the bar graphs in this figure and those proceeding it are presented as mean values +/− SEM; * *p* < 0.05.

The preliminary studies have shown that HCO_3_^−^ increased the GABA_A_R-mediated Cl^−^ influx in the neurons [40]. In our study, the SNs in the presence of HCO_3_^−^ (25 mM) showed increased Cl^−^ influx in response to the first application of GABA (100 μM) with a maximum peak of fluorescence changes of 13.7 ± 2.1% (*n* = 5) (Figure 2A). Reapplication of GABA (100 μM) revealed a decrease in the GABA_A_R-mediated Cl^−^ influx by two times with a maximum peak of fluorescence changes of 6.5 ± 1.0% (*n* = 5). Previously, it was shown that the effect of ATP and γPAs on GABA_A_R-mediated Cl^−^ transport in the neurons depends on the presence of HCO_3_^−^ in the experimental medium [22,23,24]. To probe whether the HCO_3_^−^ can regulate the GABA_A_R function, we studied the effect of γPAs on the GABA_A_R-mediated Cl^−^ influx in the presence of a physiological concentration of HCO_3_^−^ (25 mM) (Figure 2A). Here, γPAs eliminated the GABA-mediated Cl^−^ influx into neurons at the first application and activated it approximately two times on reapplication of the agonist (Figure 2A). Bicuculline completely suppressed the first peak of the GABA-mediated fluorescence changes, confirming the receptor-dependent method of mediator action (Figure 2B). In contrast, at reapplication of the mediator, bicuculline had no significant influence (7.5 ± 0.8%, *n* = 6) on the Cl^−^ influx in the SNs (Figure 2C). Similar to bicuculline’s effect, NEM (300 μM) inhibited (1.3 ± 1.2%, *n* = 6) on the first application and had no effect (6.5 ± 1.0%, *n* = 6) on repeated application of GABA (Figure 2D). Meanwhile, in the presence of VO_4_^3^^−^ (20 μM) in the experimental medium, the NEM effect was eliminated in the first case (Figure 2D) and unchanged at reapplication of GABA (Figure 2E).

### 2.3. ATP-Dependent Recovery of GABA_A_R-Mediated pH_i_ Changes

**Figure 3:**
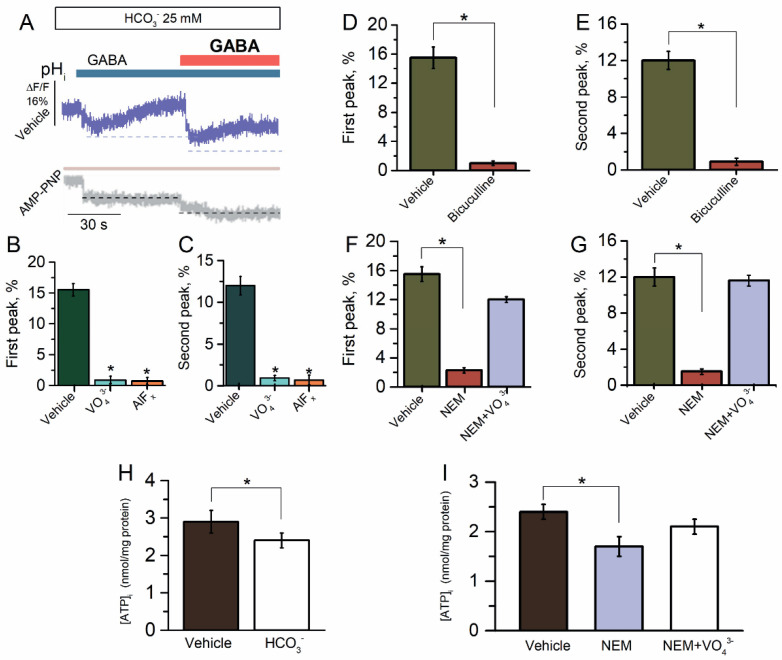
Reapplication of GABA did not reveal the desensitization of GABA_A_R-mediated HCO_3_^−^ outflux. (**A**–**C**) Representative images of BCECF fluorescence changes in SNs recorded in response to first application of GABA (100 μM) and reapplication of GABA (100 μM) in the presence of HCO_3_^−^ (25 mM) in an experimental medium, without or in the presence of VO_4_^3−^ (20 μM), AlF_x_ (20 μM), or AMP-PNP (2 mM). (**D**) Bar graph of percentage in BCECF peak fluorescence changes in response to first application of GABA (100 μM), without or in the presence of bicuculline (50 μM) (one-way ANOVA, Tukey’s test: F_(9,6)_ = 130.4, *p* = 0.000075, *n* = 6). (**E**) Bar graph of percentage in BCECF peak fluorescence changes in response to reapplication of GABA (100 μM), without or in the presence of bicuculline (50 μM) (one-way ANOVA, Tukey test: F_(9,6)_ = 58.2, *p* = 0.00005, *n* = 6). (**F**) Bar graph of percentage in BCECF peak fluorescence changes in response to first application of GABA (100 μM) without or containing NEM (300 μM), in the absence or presence of VO_4_^3−^ (20 μM) in an experimental medium (one-way ANOVA, Tukey’s test: F_(9,6)_ = 104.7, *p* = 0.0000005 or F_(9,6)_ = 4.2, *p* = 0.05, *n* = 6), respectively. (**G**) Bar graph of percentage in BCECF peak fluorescence changes in response to reapplication of GABA (100 μM) without or containing NEM (300 μM), in the absence and presence of VO_4_^3−^ (20 μM) in an experimental medium (one-way ANOVA, Tukey’s test: F_(19)_ = 53.1, *p* = 0.00005 or F_(19)_ = 0.03, *p* = 0.05, *n* = 5), respectively. (**H**) Bar graph of evaluation of [ATP]_i_ activity before and after the application of HCO_3_^−^ (25 mM) (one-way ANOVA, Tukey’s test: F_(9,6)_ = 8.9, *p* = 0.05, *n* = 5). (**I**) Bar graph of evaluation of [ATP]_i_ before and after the application of NEM (300 μM) in the absence or presence of VO_4_^3−^ (20 μM) in an experimental medium (one-way ANOVA, Tukey’s test: F_(9,6)_ = 11.0 or F_(9,6)_ = 0,005, *p* = 0.05, *n* = 5). All data in the bar graphs in this figure and those proceeding it are presented as mean values +/− SEM; * *p* < 0.05.

The rat hippocampal pyramidal neurons show an instantaneous net influx of Cl^−^ and efflux of HCO_3_^−^ in response to GABA (500 μM) application with a subsequent steady-state decrease in intracellular pH (pH_i_) of 0.2 to 0.5 [41,42]. To explore the GABA_A_R-mediated HCO_3_^−^ outflux, the SNs initially were loaded with dye for pH_i_ (BCECF) before being exposed to GABA (100 μM). Since at reapplication of the same concentration of GABA (100 μM) [43,44], the GABA_A_Rs are permeable to HCO_3_^−^, we studied the change in the pH_i_ in the SNs after the first and repeated application of GABA (100 μM) in the presence of HCO_3_^−^ (25 mM). Here, similar results were observed, where SNs quickly demonstrated a pH_i_ decrease for both the first and repetitive application of the agonist with a maximum peak of fluorescence changes of 15.5 ± 1.5% (*n* = 6) and 12.0 ± 1.0% (*n* = 6), respectively (Figure 3B,C). These data showed the absence of desensitization and only appeared in the process of resensitization. To further elucidate the cause of pH_i_ changes by GABA application, we added bicuculline to the experimental medium. Bicuculline (50 μM) inhibited the GABA-mediated HCO_3_^−^ outflux to first and repeated application of the agonist (Figure 3D,E). We added the γPAs in the experimental medium to assess the role of ATPase in the recovery of GABA_A_R-mediated HCO_3_^−^ outflux. All γPAs eliminated the GABA-mediated pH_i_ changes in the first and repeated application of the agonist (Figure 3A–C).

When exposed to NEM (300 μM), the GABA-mediated pH_i_ changes were significantly eliminated for both the first and repeat application of GABA (Figure 3F,G). However, the NEM effect did not appear in the presence of VO_4_^3−^ (20 μM), thus indicating the energy-dependent method of action (Figure 3F,G). These data suggest that NEM and HCO_3_^−^ have similar binding sites that are close to the ATP-hydrolyzing center. In order to ascertain this hypothesis, we examined the influence of HCO_3_^−^ and NEM on [ATP]_i_. In the control samples, the concentration of [ATP]_i_ was 3.0 nmol/mg protein and the addition of HCO_3_^−^ (25 mM) induced a decrease in [ATP]_i_ by 29.2 ± 1.0% (*n* = 6) (Figure 3H). Moreover, as shown in Figure 3I, the addition of NEM in the experience medium also resulted in a decline in [ATP]_i_ by 37.6 ± 1.2% (*n* = 5). However, the influence of NEM on [ATP]_i_ did not appear in the presence of VO_4_^3−^ (20 μM).

### 2.4. β3 Subunit Is Responsible for GABA_A_R Resensitization in HEK 293FT Cells

**Figure 4:**
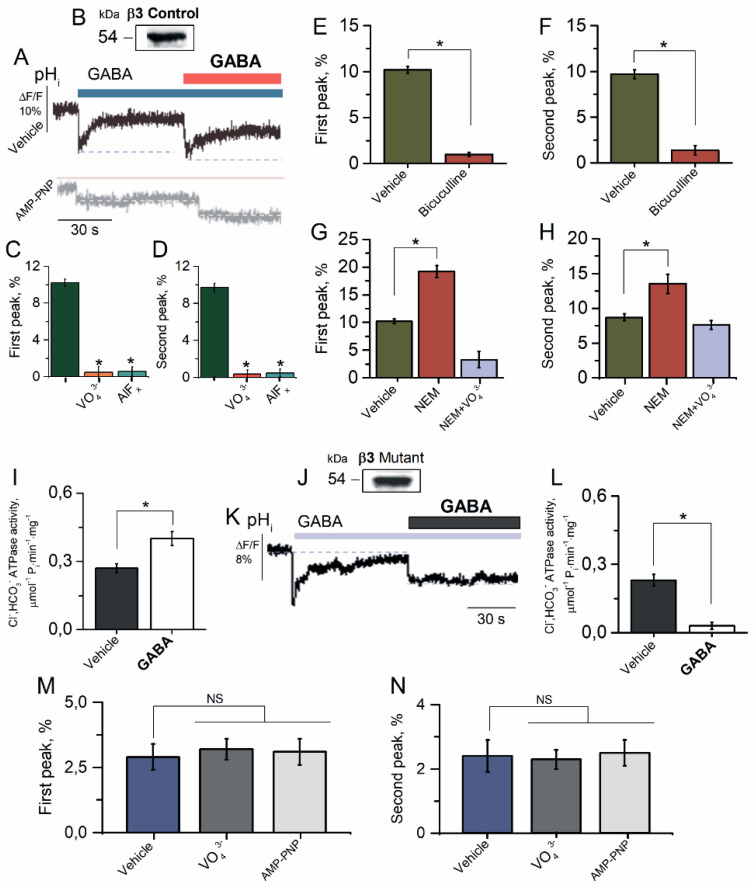
β3 subunit is responsible for GABA_A_R resensitization in HEK 293FT cells. (**A**) Representative images of BCECF fluorescence changes in HEK 293FT cells expressing β3 GABA_A_R isoform and (**B**) Western blot analysis binding of VLPs with antibody against GABA_A_R β3 subunit after expression of GABA_A_R β3 subunit in HEK 293FT cells. (**C**) Bar graph of percentage in BCECF peak fluorescence changes in HEK 293FT cells expressing β3 GABA_A_R isoform recorded in response to first application of GABA (100 μM), without or in the presence of VO_4_^3−^ (20 μM) or AlF_x_ (20 μM) (one-way ANOVA, Tukey’s test: F_(19)_ = 5.0, *p* = 0.05 or F_(19)_ = 2.18, *p* = 0.05, *n* = 5). (**D**) Bar graph of percentage in BCECF peak fluorescence changes in HEK 293FT cells expressing β3 GABA_A_R isoform recorded in response in response to second application of GABA (100 μM), without or in the presence of VO_4_^3−^ (20 μM) or AlF_x_ (20 μM) (one-way ANOVA, Tukey’s test: F_(19)_ = 5.0, *p* = 0.05 or F_(19)_ = 2.18, *p* = 0.05, *n* = 5). (**E**) Bar graph of percentage in BCECF peak fluorescence changes in HEK 293FT cells expressing β3 GABA_A_R isoform recorded in response to first application of GABA (100 μM), without or in the presence of bicuculline (50 μM) (one-way ANOVA, Tukey’s test: F_(19)_ = 192.5, *p* = 0.000001, *n* = 5). (**F**) Bar graph of percentage in BCECF peak fluorescence changes in HEK 293FT cells expressing β3 GABA_A_R isoform recorded in response in response to second application of GABA (100 μM), without or in the presence of bicuculline (50 μM) (one-way ANOVA, Tukey’s test: F_(19)_ = 268.0, *p* = 0.0000005, *n* = 5). (**G**) Bar graph of percentage in BCECF peak fluorescence changes in HEK 293FT cells expressing β3 GABA_A_R isoform recorded in response to first application of GABA (100 μM) without or containing NEM (300 μM), in the absence and presence of VO_4_^3−^ (20 μM) in an experimental medium (one-way ANOVA, Tukey’s test: F_(19)_ = 36.5, *p* = 0.001 or F_(19)_ = 29.0, *p* = 0.005, *n* = 5), respectively. (**H**) Bar graph of percentage in BCECF peak fluorescence changes in HEK 293FT cells expressing β3 GABA_A_R isoform recorded in response to first and repeat application of GABA (100 μM) without or containing NEM (300 μM), in the absence and presence of VO_4_^3−^ (20 μM) in an experimental medium (one-way ANOVA, Tukey’s test: F_(19)_ = 5.0, *p* = 0.05 or F_(19)_ = 2.18, *p* = 0.05, *n* = 5), respectively. (**I**) Bar graph of evaluation of Cl^−^, HCO_3_^−^ ATPase activity before and after the application of GABA (100 μM) in HEK 293FT cells expressing β3 GABA_A_R isoform (one-way ANOVA, Tukey’s test: F_(6,9)_ = 7.84, *p* = 0.005, *n* = 7). (**J**) Western blot analysis binding of VLPs with antibody against GABA_A_R β3 subunit after expression of mutant (C313A) GABA_A_R β3 subunit in HEK 293FT cells. (**K**) Representative images of BCECF fluorescence changes in HEK 293FT cells expressing mutant β3 GABA_A_R isoform recorded in response to first application of GABA (100 μM) and reapplication of GABA (100 μM) in the presence of HCO_3_^−^ (25 mM) in an experimental medium. (**L**) Bar graph of evaluation of Cl^−^, HCO_3_^−^ ATPase activity before and after the application of GABA (100 μM) in HEK 293FT cells expressing mutant β3 GABA_A_R isoform (one-way ANOVA, Tukey’s test: F_(0,02)_ = 7.84, *p* = 0.005, *n* = 7). (**M**) Bar graph of percentage in MQAE peak fluorescence changes in response to first application of GABA (100 μM) in the absence and presence of VO_4_^3−^ (20 μM) or AMP-PNP (2 mM) in an experimental medium (one-way ANOVA, Tukey’s test, *n* = 5), respectively. (**N**) Bar graph of percentage in MQAE peak fluorescence changes in response to reapplication of GABA (100 μM) in the absence and presence of VO_4_^3−^ (20 μM) or AMP-PNP (2 mM) in an experimental medium (one-way ANOVA, Tukey’s test, *n* = 5), respectively. All data in the bar graphs in this figure and those proceeding it are presented as mean values +/− SEM; * *p* < 0.05.

Previously, we have shown that only the GABA_A_R β3 subunit, in contrast to α2 or γ2 subunits, participates in the GABA-mediated or ATP-dependent Cl^−^ transport [27]. To examine whether the β3 subunit is also involved in the HCO_3_^−^ transport, we studied the properties of the GABA_A_R-mediated HCO_3_^−^ outflux in the HEK 293FT cells expressing the homomeric GABA_A_R β3 isoform (Figure 4A). HEK 293FT cells expressing the GABA_A_R β3 isoform showed one band in the VLPs with a molecular weight of approximately ~54 kDa that was bound to antibodies against the GABA_A_R β3 subunits (Figure 4B). The HEK 293FT cells in the presence of HCO_3_^−^ (25 mM) in the experience medium showed a rapid pH_i_ decrease in the both first and repetitive applications of the agonist with a maximum peak of fluorescence changes of 10.2 ± 0.4% (*n* = 5) and 9.7 ± 0.5% (*n* = 5) (Figure 4C,D), respectively, and with a subsequent rapid (~40 s) recovery of [HCO_3_^−^]_i_ (Figure 4A). γPAs completely eliminated the GABA-mediated fluorescence changes in the first application and at reapplication of GABA (100 μM) (Figure 4A,C,D). Bicuculline (50 μM) completely eliminated the fluorescence changes, recorded in response to first and reapplication of GABA (100 μM) in an experimental medium (Figure 4E,F).

NEM (300 μM) activated the HCO_3_^−^ outflux at the first and repeated application of GABA with a maximum peak of fluorescence changes of 19.2 ± 1.1% ***(****n* = 5) and 13.5 ± 1.4% (*n* = 5), respectively (Figure 4G,H). The NEM effect did not appear in the presence of VO_4_^3^^−^ (20 μM) assuming the involvement of the ATP-hydrolyzing system in the process of [HCO_3_^−^]_i_ recovery. To assess the role of ATPase in the consuming of [ATP]_i_, we studied the effect of GABA on the Cl^−^, HCO_3_^−^ ATPase activity. As shown in Figure 4I, the control GABA_A_R β3 isoform showed Cl^−^, HCO_3_^−^ ATPase activity of 0.27 ± 0.2 μmol^−1^ P_i_ min^−1^ mg^−^^1^. The addition of GABA (100 μM) induced increased enzyme activity by one and a half times.

To examine the contribution of the β3 subunit in the processes of resensitization, we selected a mutation nearby the desensitization-gate, which was previously found to specifically alter the desensitization kinetics and amplitude [10,17,18]. Specifically, a single cysteine residue (C313) in the M3 domain was conserved in all GABA_A_R β subunits and demonstrated a specific response to modulation by oxidizing agents [45]. To test whether this cysteine residue formed at least in part the molecular basis for NEM modulation, this residue was mutated to alanine in the β3 subunit (C313A). HEK 293FT cells expressing the mutant GABA_A_R β3 isoform showed one band in the VLPs with a molecular weight of approximately ~54 kDa that was bound to antibodies against the GABA_A_R β3 subunits, respectively (Figure 4J). In contrast to the control receptor isoform, the homomeric mutant GABA_A_R β3 (C313A) isoform displayed the GABA-mediated recovery of pH_i_ (8.9 ± 0.7%) in the first case and at the second application of GABA, showing desensitization (Figure 4K). The mutant GABA_A_R β3 isoform also possessed Cl^−^, HCO_3_^−^ ATPase activity (0.23 ± 0.01 μmol^−1^ P_i_ min^−1^ mg^−1^), and an exposition with GABA (100 μM) only decreased the enzyme activity (Figure 4L). Meanwhile, in the HEK 293FT cells expressing the homomeric GABA_A_R β3 isoform, GABA (100 μM) in both the first and repetitive application induced Cl^−^ influx in cells, but it was not statistically significant (Figure 4M,N). The maximal peak of fluorescence changes for the first and repeated application was 2.9 ± 0.8 (*n* = 5) and 2.3 ± 0.9 (*n* = 5), respectively, and that had few changes in the presence of VO_4_^3^^−^ (20 μM) or AMP-PNP (2 mM).

### 2.5. The Reconstituted Cl^−^, HCO_3_^−^ ATPase Responsible for GABA_A_R Resensitization

We followed-up on our results showing that the β3 subunit alone sufficed to show ATPase activity by transfecting HEK 293FT cells with plasmid vectors containing GABA_A_R cDNA of the β3 subunit bound to a His-tag fusion protein [27]. These studies showed that reconstituted GABA_A_Rs can participate in both the influx and efflux of Cl^−^. We purified and reconstituted the enzyme and investigated the ATP-dependent Cl^−^-transport in proteoliposomes containing the embedded protein and the fluorescent dye MQAE. To understand how the β3 subunit might affect GABA_A_R resensitization, the proteoliposomes after addition of 2 mM Mg^2+^-ATP were exposed with the agonist.

**Figure 5:**
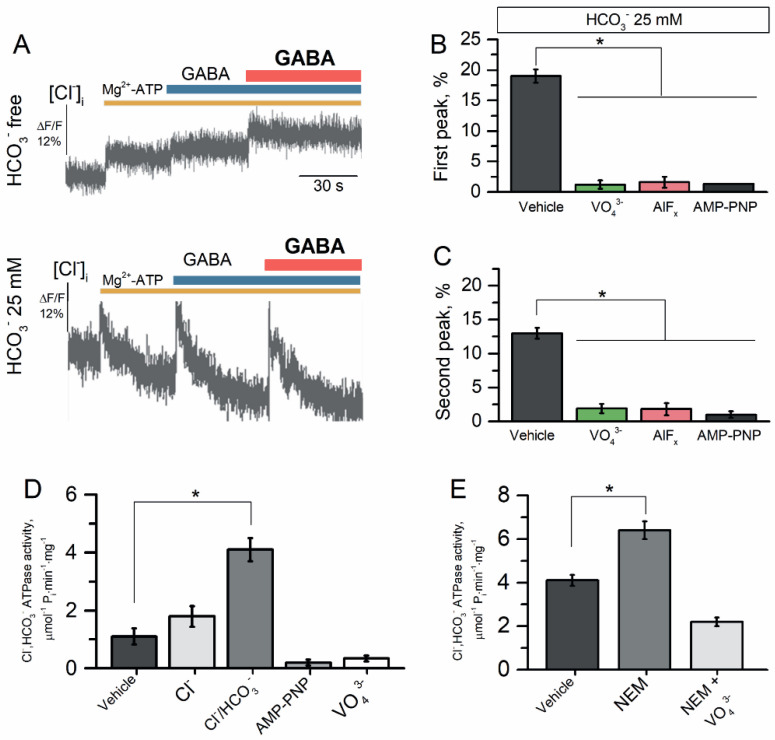
The reconstituted Cl^−^, HCO_3_^−^ ATPase responsible for GABA_A_R resensitization. (**A**) Representative images of MQAE fluorescence changes in the proteoliposomes recorded in response to addition of Mg^2+^-ATP (2 mM) before or after first and repeated application of GABA (100 μM) in an experimental medium in the presence or absence of HCO_3_^−^ (25 mM). (**B**) Bar graph of percentage in MQAE peak fluorescence changes in the proteoliposomes recorded in response to first application of GABA (100 μM), without or in the presence of VO_4_^3−^ (20 μM), AlF_x_ (20 μM), or AMP-PNP (2 mM) in an experimental medium (one-way ANOVA, Tukey’s test: F_(18,5)_ = 239.0, *p* = 0.0000005, *n* = 4). (**C**) Bar graph of percentage in MQAE peak fluorescence changes in the proteoliposomes recorded in response to first application of GABA (100 μM), without or in the presence of VO_4_^3−^ (20 μM), AlF_x_ (20 μM), or AMP-PNP (2 mM) in an experimental medium (one-way ANOVA, Tukey’s test: F_(18,5)_ = 65.0, *p* = 0.00001, *n* = 4). (**D**) Bar graph of evaluation of Cl^−^ ATPase or Cl^−^, HCO_3_^−^ ATPase activity before and after addition of AMP-PNP (2 mM) or VO_4_^3−^ (20 μM) (one-way ANOVA, Tukey’s test: F_(9,6)_ = 7.84, *p* = 0.005, *n* = 6). (**E**) Bar graph of evaluation of Cl^−^, HCO_3_^−^ ATPase activity before and after the application of NEM (300 μM) without or in the presence of VO_4_^3−^ (20 μM) (one-way ANOVA, Tukey’s test: F_(9,6)_ = 7.84, *p* = 0.005, *n* = 6). All data in the bar graphs in this figure and those proceeding it are presented as mean values +/− SEM; * *p* < 0.05.

As shown in Figure 5A (top image), addition of 2.0 mM Mg^2+^-ATP to the HCO_3_^−^-free medium resulted in a short-term (1–30 s) increase in the flow of Cl^−^ into the proteoliposomes of 5.5 ± 0.4% (*n* = 4); this influx reached a plateau after 40 s of incubation. The first and repeated application of GABA (100 μM) did not change the Cl^−^ influx to proteoliposomes in the HCO_3_^−^-free medium. Meanwhile, as shown in Figure 5A (bottom image), in the presence of HCO_3_^−^ (25 mM) in the experimental medium, addition of Mg^2+^-ATP (2.0 mM) increased the Cl^−^ influx into the proteoliposomes during the short-term (1–30 s); this Cl^−^ influx reached a plateau after 30 s of incubation with a maximal peak of the fluorescence changes (19.5 ± 0.7%, *n* = 4).

The first addition of GABA (100 μM) to the incubation medium caused a short-term (~1 s) efflux of Cl^−^ from the proteoliposomes with a maximal peak of fluorescence change of 18.0 ± 1.3% (*n* = 4) that was quickly restored and reached a plateau during ~60 s of incubation (Figure 5A). Repetitive application of GABA (100 μM) also caused a short-term (~1 s) efflux of Cl^−^ from the proteoliposomes with a maximal peak of fluorescence change of 13.0 ± 0.5% (*n* = 4) that was quickly restored and reached a plateau during ~60 s. Both the first and repetitive application of the agonist were inhibited by γPAs, showing the involvement of ATPase activity (Figure 5B,C). To examine the role of ATPase in the GABA-mediated Cl^−^ influx into proteoliposomes, we studied its activity in the absence or presence of γPAs. The reconstituted receptor showed Cl^−^, HCO_3_^−^ ATPase activity in 4.1 ± 0.3 μmol^−1^ P_i_ min^−1^ mg^−1^ (Figure 5D). In addition, AMP-PNP (2 mM) and VO_4_^3^^−^ (20 μM) suppressed the Cl^−^, HCO_3_^−^ ATPase activity. An application of NEM (300 μM) increased the enzyme activity (6.4 ± 0.4 μmol^−1^ P_i_ min^−1^ mg^−1^) and this effect was eliminated by the addition of VO_4_^3−^ (20 μM) (Figure 5E). 

## 3. Discussion

**Figure 6:**
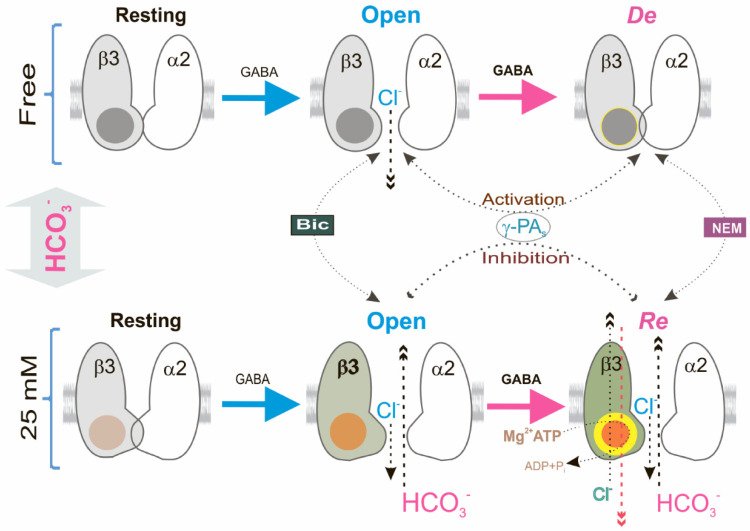
Bicarbonate preferentially stabilized GABA_A_R via ATPase performance. Model showing four basic conformation states that depict the GABA_A_R function: a resting state; an open state; a desensitization state; and a resensitization state. In an HCO_3_^−^-free experimental medium, fist application of GABA shifted the equilibrium from resting state to high-affinity open state, and repeat application of agonist induced GABA_A_R desensitization. In contrast, in the presence of HCO_3_^−^ (25 mM) in an experimental medium, first application of GABA induced the changes in the conformation of channel and shifted the equilibrium from resting state to high-affinity open state, whereas the reapplication of agonist induced GABA_A_R resensitization by β3-coupled ATPase operation.

Although the involvement of kinases and other molecules in the desensitization and slowdown of deactivation of GABA_A_R-mediated currents has been shown [9,20], the role of the ATP-hydrolyzing system in these processes is less explored. Currently, three fundamental states have been established that depict the GABA_A_R channel function at sustained or repeated application of agonist binding: resting, open, and desensitized states [2,16]. Here, by repeated use of the agonist, we also observed three forms of GABA_A_R-mediated Cl^−^ influx in the neurons that vary in their sensitivity to γPAs (Figure 6). The open GABA_A_R state, in contrast to the desensitization state, was activated by γPAs (Figure 1) which is similar to data from other studies. Specifically, blockers of phosphatases (vanadate or okadaic acid) in the concentration of 100 μM [20] as well as AMP-PNP (1 mM) [21,25] activated the GABA_A_R-mediated Cl^−^ current via changes of processes of phosphorylation and dephosphorylation [46]. However, we found that in the presence of physiological concentrations of HCO_3_^−^, blockers as well as AMP-PNP inhibited the open state of the GABA_A_Rs that were shown to take part in the ATP-consuming system in the processes of desensitization and resensitization (Figure 2). This assumption was also supported by the data about changes in [ATP]_i_ in the presence of a physiological concentration of HCO_3_^−^ and the alkylating agent NEM (Figure 3). AMP-PNP, which traps the magnesium nucleotide within the ATP-hydrolyzing site [30], effectively eliminated the GABA_A_R-mediated Cl^−^/HCO_3_^−^ fluxes. Similar conclusions were drawn from experiments in which the GABA_A_R conductance was maintained by the addition of Mg^2+^-ATP (2 mM) not revealed in the presence of non-hydrolysable ATP analogs (AMP-PNP or AMP-PCP) [22,23,24,25].

GABAergic neurotransmission has the unique property of ionic plasticity, rooted in the short-term and long-term changes in the neuronal intracellular concentrations of chloride ([Cl^−^]_i_) and bicarbonate ([HCO_3_^−^]_i_) ions [47,48]. Although GABA_A_Rs are mainly permeable to Cl^−^, they also show a significant permeability to HCO_3_^−^ (at a ratio of HCO_3_^−^ to Cl^−^ of ~0.2–0.4). In mature neurons, the [Cl^−^]_i_ is low, the neuronal equilibrium potential for chloride (E_Cl_^−^) is negative, and GABA_A_R activation triggers Cl^−^ influx and subsequent hyperpolarization or depolarization that depends not only on [Cl^−^]_i_ but also on [HCO_3_^−^]_i_ [49,50]. Experimental studies have demonstrated that massive GABAergic stimulation can shift E_GABA_ from hyperpolarizing toward depolarizing and even excitation [42,44]. In contrast to Cl^−^, much less is known about the role of HCO_3_^−^, which despite a lower permeability, invariably flows outward after significant GABA_A_R activation with a consequent dramatic fall in intracellular pH (pH_i_) [41,50,51,52]. Moreover, it was shown that the reapplication of GABA (100 μM) does not reveal the desensitization of bicuculline-inhibited and GABA_A_R-mediated HCO_3_^−^ outflux in in situ experiments [43,50]. Here, we showed that the presence of HCO_3_^−^ not only increased the [Cl^−^] but also reduced pH_i_ after HCO_3_^−^ outflow via the GABA_A_R pore. We also did not find that the desensitization of GABA_A_R-mediated HCO_3_^−^ outflux at the second application of the agonist (Figure 3). In addition, such recovery of [HCO_3_^−^]_i_ after GABA_A_R-mediated activity occurring at ~40 s is consistent with data on the recovery of GABA_A_R-mediated conductance after desensitization at ~30 s [41,51].

Unlike the various plasmalemmal ATPases that use the ATP energy for actively transporting ions against an electrochemical gradient [53], GABA_A_Rs have passive conduits for anions [1]. Our studies also demonstrated that the GABA_A_Rs have passive permeability for Cl^−^ or HCO_3_^−^ (Figure 1 and Figure 3). The homomeric β3 GABA_A_R subtype operates in two different modes, which was shown before in the presence of HCO_3_ as a GABA-gated ion channel or a primary-active ATP-consuming transporter [27]. Such properties are characteristic of several systems, including intensively studied systems such as ATP-binding cassette importers [54] or exporters [55] inhibited by high concentrations (≥100 μM) of vanadate or okadaic acid [56,57]. However, detailed mechanistic insights into the role of ATP consumption in the function of these systems are lacking and most probably involves in channel gating [58]. P-type ATPases are characterized by the formation of transit high-energy acyl phosphate intermediates, where ATP is bound at the catalytic site as a planar structure in a complex with water and Mg^2+^ in a dephosphorylation transition state-like conformation that is inhibited by low concentrations (≤20 μM) of VO_4_^3−^ or metal fluoride (MeF_x_) complexes [56,57]. Previously, we showed that the GABA_A_R-coupled ATPase can form a phosphate intermediate that is dephosphorylated in the presence of anions and inhibited by low concentrations of VO_4_^3−^ (≤20 μM) [28]. Phosphorylation of the β3 subunit was seemingly an intermediate step required for energy transduction and displacement of bound anions before hydrolytic cleavage of P_i_ during the resensitization state (Figure 1 and Figure 2). In addition, the NEM effect on the GABA_A_R-mediated Cl^−^ or HCO_3_^−^ fluxes was eliminated by vanadate in the presence of HCO_3_, which denoted a close site of localization of the ATP-hydrolyzing center and cysteine residue (C313) in the M3 domain of the β3 subunit (Figure 6). This line of reasoning confirms data where the chimeric isoform did not show the desensitization of the GABA_A_R-mediated Cl^−^ flux but only the resensitization.

Although stereotypical synaptic GABA_A_Rs are composed of two α, two β, and one γ subunit [59], the functional properties of GABA_A_Rs are ensured by the mandatory inclusion of the β subunits and primary β3 subunit [60]. Furthermore, the β3 subunit possesses a set of functional and pharmacological properties that distinguish it from other β subunits [28,61]. These observations lend a new physiological significance to the β3 subunit in the manifestation of resensitization of GABA_A_R via the involvement of the ATPase. The changing [HCO_3_^−^]_i_ is the major determinant of the appearance of GABA_A_R resensitization, whereas the changing [Cl^−^]_i_ in the bicarbonate-free medium plays a vital role in their desensitization that is similar to data of other studies. In the particular, it was found that the changing [Cl^−^]_i_ is the major determinant of GABA_A_R-mediated current decay in the presence of an agonist [11,62]. Overall, we describe a new investigation of the role of HCO_3_^−^ in the GABA_A_R function and revealed a molecular mechanism whereby a massive activation of GABA_A_Rs can strengthen or reduce the inhibition via GABAergic synapses. In this context, given the current structural and kinetic evidence, the physiological role of the ATPase appears to be essential due to its involvement in the long-lived conformational change of receptors, transduced from the external to the internal faces of the plasma membrane upon the binding of an agonist. However, structural studies are required to establish precisely what kind of molecular rearrangements take place in the structure of the β3 subunit during the transition from desensitization to resensitization.

## 4. Materials and Methods

### 4.1. Animals and Housing

Animal experiments were carried out using adult male Wistar rats purchased from the Institute of General Pathology and Pathophysiology vivarium and weighing 130–160 g at the time of arrival unless otherwise stated. Rats were always group-housed (5 per cage) and maintained in a temperature-controlled environment (23 ± 1) on a 12:12 h light-dark cycle and had access to food and water ad libitum. We performed all manipulations on animals in accordance with EU directive 2010/63/EU and according to the principles expressed in the Declaration of Helsinki revised by WMA, Fortaleza, Brazil, 2013, and the Rules of Good Laboratory Practice in the Russian Federation approved by Order N 199_H_ (1 April 2016) of the Ministry of Health Care, under supervision of the Ethics Committee of the Institute of General Pathology and Pathophysiology (project approval protocol No 3 of 18 08 2021; the final approval protocol No 1 of 03 03 2022).

### 4.2. Synaptoneurosomes (SNs) Preparation

SNs were prepared from whole brains of wild-type from freshly dissected forebrains (mostly cortex) (~200–400 mg wet weight) as previously described [63]. Briefly, rats were quickly decapitated using a guillotine, brains were removed and placed in an ice-cold, balanced salt solution (BSS) containing 135 mM NaCl, 1 mM KCl, 0.8 mM MgCl_2_, 0.5 mM KH_2_PO_4_, 10 mM glucose, 0.1% bovine serum albumin (BSA), 10 mM Hepes-Tris (pH 7.3), and a protease inhibitor (A32955, Thermo Fisher Scientific, USA). The brain was cut into small pieces (2–3 mm) and manually homogenized (6 strokes) with a loosely fitting glass-Teflon homogenizer. The homogenate was passed through a nylon mesh (80 μm), and the filtrate was subsequently passed through a cellulose nitrate filter (8 μm) followed by centrifugation at 1000× *g* for 15 min. The pellet was washed once in BSS and centrifuged. All the procedures were performed at 4 °C. Sodium chloride (7647-14-5), potassium chloride (7447-40-7), magnesium chloride (7786-30-3), potassium phosphate monobasic (7778-77-0), BSA (9048-46-8), and D-(+)-Glucose (50-99-7) were obtained from Merck, (Branchburg, NJ, USA).

### 4.3. Plasma Membrane (PM) Preparation

PMs were prepared from control HEK 293FT cells and various GABA_A_R variants were detached using Hanks’ balanced salt solution (Gibco, Waltham, MA, USA) without divalent cations (i.e., trypsin was not used), and the cells were centrifuged at 300× *g* for 3 min. The HEK 293FT cells or brain (mostly cortex) were homogenized in an ice-cold buffer containing 0.3 M sucrose, 0.5 mM EDTA-Tris, HEPES-Tris, 10 mM (pH 7.3), and protease inhibitor cocktail tablets (A32955, Thermo Fisher Scientific, Waltham, MA, USA), and centrifuged at 10,000× *g* for 15 min at 4 °C, after which the pellet was discarded. The supernatant was centrifuged for 1 h at 150,000× *g* and the resulting pellets were resuspended in 20 mM HEPES-Tris pH 7.3. This plasma membrane-enriched preparation was used for further measurements of the enzyme activity. Ethylenediaminetetraacetic acid (60-00-4), 4-(2-hydroxyethyl)-1-piperazineethane-sulfonic acid (HEPES), and Tris(hydroxymethylamino-methane (77-86-1) were obtained from Merck (USA).

### 4.4. Cell Cultures and Transfection

For the expression homo- or heteromeric GABA_A_R ensembles, human embryonic kidney 293FT cells (American Type Culture Collection) were used. The cells were purchased from Invitrogen (USA) as part of the Membrane Pro™ Functional Protein Expression System (A11667), and the cell line identity was not further authenticated. The cells were grown and maintained in an incubator (Sanyo, Osaka, Japan) at 37 °C in a humidified atmosphere with 5% CO_2_, in DMEM media (41965-039, Gibco, Inchinnan, UK) supplemented with of 0.1 mM MEM NEAA (11140035, Gibco, Inchinnan, UK), 4 mM L-glutamine, 1 mM sodium pyruvate, 4.5 g/L D-glucose (15023021), and 10% FBS ((10270-106, Gibco, Germany) until the 20th passage, as suggested by the vendor. HEK 293FT cells were transfected by Lipofectamine TM 2000 or 3000 (Invitrogen, Thermo Fisher Scientific, Waltham, MA, USA and Lithuania) transfection reagents according to the manufacturer’s instructions. Cells were harvested and analyzed 24 h after transfection. For transfection procedures and virus-like particle (VLP) production, the same growth medium with decreased FBS content up to 4% was used according to the manufacturer’s recommendations. Geneticin G418 sulphate (11811031, Invitrogen, Waltham, MA, USA) was present in the growth medium at a concentration of 500 mg/mL constantly except during the transfection. The cells were subcultured at confluence by treatment with 0.05% trypsin and 0.02% EDTA in PBS. For selection purposes and improving the yield of VLPs, the transfection medium was removed after 24 h and a fresh growth medium with 10 µg/mL blasticidin (R21001 Gibco, USA) was added. Transfected cells and VLPs were collected and analyzed 24–48 h after transfection.

### 4.5. Molecular Biology

The genes encoding the full-length rat GABA_A_R β3 subunit were amplified by PCR from the cDNA library (Evrogen, Moscow, Russia) using gene-specific primers with Kozak sequence at the 5′ end of the forward primer based on “GenBank: NM_017065.1” sequences. The PCR products were cloned into the pEF6/V5-His TOPO TA vector (K961020, Invitrogen, Waltham, MA, USA) separately and verified by DNA sequencing. Each vector was amplified using *E. coli* TOP10 strain in LB medium supplemented with 20 µg/mL ampicillin. Isolation and purification of plasmids were performed with PureYieldTM Plasmid Miniprep System (Promega, Madison, WI, USA) and Plasmid Midiprep 2.0 (Evrogen, Moscow, Russia). The sterilization of plasmids was implemented via 0.22 µm filtration. The concentration of plasmids was evaluated on spectrophotometer NanoDrop 1000 (Thermo Fisher Scientific, Waltham, MA, USA). The quality validation of cloning and growth was performed additionally through enzymatic restriction by XbaI and BamHI in BamHI buffer (Thermo Fisher Scientific, Waltham, MA, USA), and the following electrophoresis in 1% agarose gel.

### 4.6. Transfection

The typical transfection procedure of GABA_A_R subunit-containing constructs for the subsequent biochemical, spectrofluorometric, and Western blot analyses was as follows. Approximately 5 × 105 HEK293FT cells were suspended in 8 mL DMEM, plated into a 90-mm culture dish, and maintained 24 h approximately until 50% to 90% of the confluence. Then, 5 μg of plasmid DNA (β3 alone) was added combined with Lipofectamine^®^ 3000 Reagent (Invitrogen, Thermo Fisher Scientific, Waltham, MA, USA) in Opti-MEM^®^ I (1×) + GlutaMAXTM-I medium (51985-026, Gibco, Inchinnan, UK) accordingly the manufacturers’ recommendations. For microscopy, the cells were plated in 35 mm dishes and were incubated with a proportional amount of reagents and vectors.

### 4.7. VLP Production

For VLP production, GABA_A_R subunit-containing constructs were transfected together with Membrane Pro™ Reagent (Invitrogen, Waltham, MA, USA) amenably. Transfected HEK293FT started to bud off VLPs from the cell membrane approximately 24 h after transfection. The harvesting procedure was executed in conformity with manufacturer’s recommendations. Briefly, the VLP-containing medium was mixed with Membrane Pro™ Precipitation Mix in the ratio of 5 to 1, where 5 refers to the medium. Then, the mix was incubated at 4 °C overnight. After incubation, VLPs were pelleted by centrifugation at 5500× *g* for 30 min and resuspended in HEPES buffer for subsequent analysis or stored at −80 °C.

### 4.8. Cl^−^ and HCO_3_^−^-Transport Assays

Cl^−^-sensitive fluorescent dye MQAE (N-(Ethoxycarbonyl-methyl)-6-Methoxyquinolinium Bromide) or BCECF, AM (2′,7′-Bis-(2-Carboxyethyl)-5-(and-6)-Carboxyfluorescein, Acetoxy methyl Ester) were obtained from Thermo Fisher Scientific, USA (E3101) or B1170, respectively. A stock solution was prepared in H_2_O, aliquoted, and stored in the freezer (−20 °C) protected from light. For cases in which fluorescence measurements were conducted, the HEK 293FT cells or SNs with loaded dye were stored in an opaque test tube at RT or 4 °C. Sodium bicarbonate (144-55-8), γ-aminobutyric acid, GABA (56-12-2), and bicuculline (485-49-4) were obtained from Sigma-Aldrich (USA), and adenosine 5′-(β,γ-imido)triphosphate lithium salt hydrate (25612-73-1) were obtained from Merck (USA). GABA_A_R-mediated Cl^−^- or HCO_3_^−^-transport was assessed by the dynamic measurements of the variations in the fluorescence intensity of Cl^−^-sensitive fluorescent dye MQAE-loaded or BCECF-loaded HEK 293FT cells or SNs using a FluoroMax^®^-4 spectrofluorometer (HORIBA Scientific Edison, Piscataway, NJ, USA), respectively. For that, the control HEK 293FT cells and various GABA_A_R β3 isoforms cells were trypsinized by adding 0.05% trypsin-EDTA solution (25200056, Gibco BRL, USA), washed PBS twice, resuspended in the BSB, and then loaded with MQAE for 1 h at 37 °C. After loading, the suspension was centrifuged at 200× *g* for 5 min at RT and kept in the aforementioned medium at RT in the opaque test tubes. For analysis, the pellet was resuspended in the BSB. Dye-loaded cells (SNS or HEK 293FT) or proteoliposomes were equilibrated in the incubation medium in the absence or presence of compounds (γPAs, bicuculline, or NEM) for about 10 min at 37 °C before initial fluorescence measurements, and then 150 μL of the suspension was added into quartz microcuvette (non-flow cell) andstirred. The GABA-mediated Cl^−^ or HCO_3_^−^ transport was initiated directly in the cuvette by an in-house solution supply system. The excitation and emission wavelengths were 350 nm and 480 nm for measurement of Cl^−^-transport or 490 nm and 535 nm for measurement of HCO_3_^−^-transport, respectively. The Δ*F*/*F* of each trial was calculated as (*F* − *F*_0_)/*F*_0_, where *F*_0_ is the baseline fluorescence signal averaged over a 25 s period (this was the control measurement) immediately before the start of the application of GABA and supplement compounds. The value of 100% was obtained as the fluorescence intensity before the application of GABA, in the absence or presence of test compounds. The maximum amplitude of GABA-mediated fluorescence responses (first peak % and second peak %) was calculated as the maximal difference in fluorescence intensity in the absence or presence of an agonist.

### 4.9. [ATP]_i_ Measurement

ATP concentrations were determined by the luciferase method. As the source of luciferase, we used the ATP-Glo luminescence kit from Biotium (Fremont, CA, USA). The ATP standards or samples of SNs were prepared in 100 μL dH_2_0 for the assay. The solution containing luciferase was reconstituted as indicated by the manufacturer and was stored in aliquots at −20 °C for at least 2 weeks. For measuring ATP, the solution was diluted 25-fold with an ATP assay mix dilution buffer. A calibration curve with 2–200 pmol of ATP was prepared by diluting an ATP stock solution (2 mM). ATP standards or samples were added to give a final volume of 500 μL. Tubes were vigorously vortexed for 3–5 s and the content was immediately placed in the 96-well black plate and then in the multilabel counter (Plate ChameleonTM V, Finland). The procedure lasted 3–5 min. D-luciferin was dissolved to a concentration of 0.4 mg/mL. Firefly luciferase was added to the ATP-Glo assay solution at a rate of 1 μL per 100 μL (25 μL luciferase per 2.5 mL). The ATP-Glo detection cocktail was prepared fresh before each use for maximum potency. The integration time of the luminescent signal was 10 s. As well, 100 μL of ATP-Glo detection cocktail was added to the sample. We mixed the mixture rapidly by pipetting. Immediately thereafter, the luciferase activity was measured at room temperature.

### 4.10. ATPase Activation Assay

The Cl^−^/HCO_3_^−^-ATPase activity in PMs of neurons or HEK 293FT cells expressing the various constructs were measured as previously described [27]. Briefly, PMs or VLPs were added to 0.5 mL of an incubation medium containing 20 mM HEPES-Tris pH 7.3, 5 mM NaCl/25 mM NaHCO_3_, and 50 mM NaNO_3_ (neutral salt) to measure enzyme activity. The enzyme preparations (~10 μg) were preincubated at 37 °C for 15 min with the relevant compounds in an incubation medium containing 20 mM HEPES-Tris pH 7.3 and 5 mM NaCl/25 mM NaHCO_3_. Preparation of the test tube with bicarbonate—NaHCO_3_ (1 mM)—was previously dissolved in HEPES (20 mM) and then added in the 20 mM HEPES-Tris buffer (pH 6.7). The reaction was started by addition of Mg^2+^-ATP 2 mM (final concentration) in an experimental medium. The Cl^−^/HCO_3_^−^-activated ATPases activity was determined as a difference in formation of inorganic phosphorus (P_i_) in the absence and in the presence of 5 mM NaCl/25 mM NaHCO_3_ in the incubation medium. Adenosine 5′-triphosphate (ATP) disodium salt hydrate (34369-07-8) and adenylyl-imidodiphosphate (25612-73-1) were obtained from Merck (USA). The concentration of inorganic phosphate (P_i_) in the incubation medium was measured by a modified method of Chen et al., (1956) [27] using a Cary 60 UV–vis spectrophotometer (Agilent, Santa Clara, CA, USA) at wavelength of 650 nm. The γ-phosphate analog, orthovanadate (VO_4_^3^^−^) (Sigma-Aldrich, St. Louis, MI, USA), was obtained by boiling the vanadate solution ([pH 10]; 10 min), and freshly boiled stock was diluted to the final concentration (pH 7.3) prior to use.

### 4.11. Western Blot Analysis

VLPs of transfected HEK 293FT cells were subjected to SDS-PAGE using the SDS-PAGE reagent starter kit (1615100 Bio-Rad, Hercules, CA, USA) and to Western blot analysis using the Pierce™ fast western blot kit (35055 Thermo Scientific, Waltham, MA, USA), ECL Plus Western Blotting Detection System Substrate (GE Healthcare, Chicago, IL, USA). Samples were SDS-treated by boiling for 5 min in a buffer consisting of 62.5 mM Tris, 10% glycerol, 5% 2-mercaptoethanol, 4% SDS, and 0.001% bromophenol blue, and then ~20 µg of total protein was loaded into SDS-PAGE. Electrophoresis parameters were: 70 V for 10 min on 4% SDS-PAGE stacking gel and 120 V for 50 min on 12.5% SDS-PAGE resolving gel. Proteins were transferred on PVDF membrane by the semi-dry method using 0.09 A/cm^2^ for 1 h. After that, membranes were incubated for 1 h in a blocking solution containing 5% milk, and then incubated at 4 °C overnight with primary anti-GABRB3 antibodies [N87/25] (ab98968, Abcam, Cambridge, UK) diluted 1:1000 with the blocking solution. After incubation, the membranes were washed with TBS-T 4 times for 15 min each, and then incubated at RT for 1 h with secondary HRP-conjugated antibodies (62-6520 Thermo Fisher Scientific, Waltham, MA, USA) diluted 1:5000 with the blocking solution. Then, the membrane was washed with TBS-T four times and the GE Healthcare ECL Plus Western Blotting Detection System (Amersham™, GE Healthcare, UK) was applied according to manufacturer’s instructions. The visualization of the bands was obtained on a Kodak Image Station 440 (USA).

### 4.12. Statistical Analysis

All the data shown in the bar graphs are presented as mean ± standard errors (SEM). This includes graphs demonstrating the median GABA_A_R-mediated events. The data were collected with a repetition during several experimental days (n) and two or three measurements were combined for the average value in each series. The one-way analysis of variance (ANOVA) followed by Tukey’s post hoc test for multiple groups comparison was performed for the data, which were assessed as normally distributed with the Shapiro-Wilk test. Results with *p* values < 0.05 were defined as statistically significant (indicated by the * symbol on graphs), and *p* values are reported in full, where appropriate, with each figure legend. Origin Pro version 9.1 for Windows (OriginLab, Northampton, MA, USA) was used for conducting of the statistical analysis and the graphic representation of the data. Origin Pro version 9.1 for Windows (OriginLab, USA) was used for conducting of the statistical analysis and the graphic representation of the data.

## Data Availability

The data presented in this study are available on request from the corresponding author.
